# Exercise‐linked improvement in age‐associated loss of balance is associated with increased vestibular input to motor neurons

**DOI:** 10.1111/acel.13274

**Published:** 2020-11-11

**Authors:** Fabienne Battilana, Stefan Steurer, Giorgio Rizzi, Ana C. Delgado, Kelly R. Tan, Christoph Handschin

**Affiliations:** ^1^ Biozentrum University of Basel Basel Switzerland

**Keywords:** aging, balance, exercise, motor control, motor neurons, proprioceptive system, training, vestibular system

## Abstract

Age‐associated loss of muscle function is exacerbated by a concomitant reduction in balance, leading to gait abnormalities and falls. Even though balance defects can be mitigated by exercise, the underlying neural mechanisms are unknown. We now have investigated components of the proprioceptive and vestibular systems in specific motor neuron pools in sedentary and trained old mice, respectively. We observed a strong age‐linked deterioration in both circuits, with a mitigating effect of exercise on vestibular synapse numbers on motor neurons, closely associated with an improvement in gait and balance in old mice. Our results thus describe how the proprioceptive and vestibular systems are modulated by age and exercise, and how these changes affect their input to motor neurons. These findings not only make a strong case for exercise‐based interventions in elderly individuals to improve balance, but could also lead to targeted therapeutic interventions aimed at the respective neuronal circuitry.

## Graphical abstract

Aging results in the degeneration of proprioceptive and vestibular wiring, associated with abnormalities in gait and balance. Exercise improves vestibular connectivity to specific motor neuron pools, and restores gait and balance.

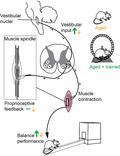

## 1 INTRODUCTION

Aging is associated with a decline in mental and motor performance as well as a loss in muscle mass and function. In addition, neuronal and muscular dysfunction in aging contributes to deterioration of body posture and balance (Fernandez et al., 2015). Combined with the age‐associated loss of muscle mass and function, termed sarcopenia, these defects promote falls, leading to fractures, hospitalization, loss of independence and admission to nursing homes (Fuller, 2000; Sturnieks et al., 2008). Every third person older than 65 falls at least once per year, contributing to the high morbidity and mortality in elderly humans (Fuller, 2000; Kannus et al., 1999). To date, no pharmacological interventions against the aging‐linked neuronal and muscular decline or the balance and motor coordination defects are known. Intriguingly however, exercise mitigates the consequences of all of these pathological events, including improvements in balance, body posture and gait in epidemiological and clinical settings (Lelard & Ahmaidi, 2015). However, the underlying neuromuscular mechanisms are only rudimentarily understood.

The sense of balance uses sensory information from vision, touch, proprioception, and vestibular input to affect motor output on the level of motor neurons and their associated spinal circuits (Sturnieks et al., 2008). The muscle spindle, sensing the stretch as well as the velocity of muscle contractions, is part of the proprioceptive circuitry. Proprioceptive sensory input generated in muscle spindles and other proprioceptors are transmitted by proprioceptive sensory neurons mono‐ or disynaptically to motor neurons (Proske & Gandevia, 2012). The vestibular system, located in the inner ear, assesses gravitational forces as well as acceleration. Vestibular sensory information is then transmitted via the vestibular ganglion and nerve to the vestibular nuclei located in the brain stem (Angelaki & Cullen, 2008). Like many proprioceptive sensory neurons, descending efferents from the lateral vestibular nuclei (LVe) form direct mono‐synaptic projections to motor neurons (Basaldella et al., 2015; Murray et al., 2018). In combination, the proprioceptive and vestibular systems are especially important for maintaining coordinative movements, body posture and balance (Angelaki & Cullen, 2008; Proske & Gandevia, 2012; Windhorst, 2007). The input of both systems is integrated by motor neurons, which then generate appropriate muscle contractions by eliciting an action potential that is transmitted to muscle fibers via the neuromuscular junction (NMJ) (Proske & Gandevia, 2012).

In sarcopenia, skeletal muscle mass and function are impaired due to pleiotropic age‐associated changes on the level of the muscles and motor nerves (Jang & Van Remmen, 2011). Moreover, detrimental effects of aging on the proprioceptive muscle spindle receptors (Kararizou et al., 2005; Liu et al., 2005; Swash & Fox, 1972), vestibular ganglions and nerves (Bergstrom, 1973; Park et al., 2001) as well as the vestibular nuclei (Alvarez et al., 1998; Lopez et al., 1997; Sturrock, 1989) have been reported in different experimental settings, resembling other age‐linked neurodegenerative events in the central and peripheral nervous system (Mentis et al., 2011; Vaughan et al., 2015).

Exercise is a powerful intervention to slow down aging pathologies (Coen et al., 2018; Vina, 2019). Of note, endurance exercise improves NMJ morphology and muscle metabolism, enhances neurogenesis, and improves learning (van Praag et al., 2005; Valdez et al., 2010; White et al., 2016). In contrast, the effects of exercise and of age on central proprioceptive and vestibular circuitries are unexplored. It thus is unclear how exercise improves age‐associated loss of balance as reported in epidemiological studies. We therefore investigated the relationship between age, the level of physical activity, the integrity of proprioceptive and vestibular systems, and age‐associated balance defects in sedentary and trained mice, respectively, at different ages and with different training interventions. The use of retro‐ and anterograde neuronal and synaptic tracing techniques combined with in vivo balance and gait testing were used to interrogate how age and exercise affect proprioception and vestibular input in these contexts. These approaches revealed a broad degenerative effect of age on the physiological parameters and the neuronal circuitry, which in regard to vestibular connectivity could be rescued by endurance training, associated with a robust improvement in gait and balance.

## 2 RESULTS

### 2.1 Exercise mitigates age‐associated changes in gait and balance

**FIGURE 1:**
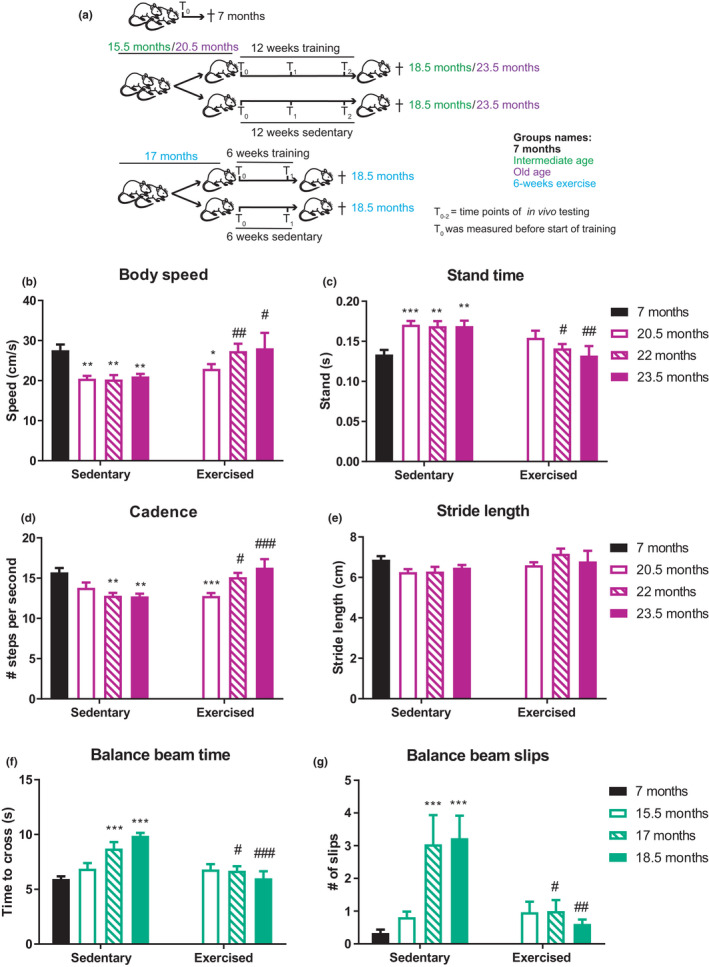
Exercise mitigates age‐associated changes in gait and balance. (a) Schematic of different groups of mice T_n_ is to show the different time points when in vivo test were repeated. (b–e) Catwalk gait analysis. Mice voluntarily walked across a glass platform where paw prints were recorded and gait assessed: Body speed (cm/s) (b), stand time (s) (c), cadence (steps/s) (d), and stride length (cm) (e) are depicted. (f, g) Balance of intermediate aged exercised and sedentary mice was assessed using a balance beam test: time to cross the length of a round balance beam (f) number of slips while traversing the beam (g). Data are displayed as mean with ±*SEM*. *n* = 6–9 animals per group. Using two‐way ANOVA, *p < 0.05; **p < 0.01; ***p < 0.001 indicate statistically significant differences between 7 months controls and aged groups (age effect), while ^#^ *p* < 0.05; ^##^ *p* < 0.01; ^###^ *p* < 0.001 indicate statistically significant differences between sedentary and exercised animals of the same age group (exercise effect)

To assess the effect of age and exercise on balance, the proprioceptive and vestibular systems, we compared young control mice to an intermediate (15.5–18.5 months) and an old age group (20.5–23.5 months), respectively, using exercise interventions of 6 and 12 weeks of length, and differences in training volume (Figure 1a). The respective ages were chosen to exclude muscle mass loss, and hence also differences in body weight (Figure S1A), as a confounding factor for the observed phenotypic changes, as demonstrated by comparing gastrocnemius (GS) (Figure S1B), tibialis anterior (TA) (Figure S1D), and extensor digitorum longus (EDL) (Figure S1D) muscle mass between the groups. Since elderly humans undergo changes in gait (Verghese et al., 2010), we first tested if aged mice would show a similar deterioration. Thus, to measure gait in aged sedentary and exercised mice, animals walked voluntarily through a Catwalk tunnel connected to a camera tracking paw prints (Figure 1b–g, Figure S1E–L, Video S1A). Old mice moved at significantly slower speed through the tunnel (Figure 1b, Figure S1D,H). This decrease in body speed was caused by an increase in stand‐time (Figure 1c, Figure S1E,I) combined with a reduction in cadence of old compared to the 7 months young control group (Figure 1d, Figure S1F,J), while stride length was only affected by age in 18.5‐month‐old mice that trained for 6 weeks (Figure 1e, Figure S1G,K). Interestingly, exercise improved body speed in all aged age groups (Figure 1b, Figure S1D,H) by decreasing stand time (Figure 1c, Figure S1E,I) and increasing cadence (Figure 1d, Figure S1F,J). In the intermediate age group, training even affected stride length (Figure S1G,K).

We next assessed the balance of aged sedentary and exercised mice in different challenges, using round and square beams, respectively (Video S1B). Balance performance deteriorated between the age of 15.5 and 17 months, both in regards to the time needed to cross the beam and the number of slips (Figure 1f,g, Figure S2A–D). In all age groups and tests, exercise significantly improved the time to cross, statistically indistinguishable from the levels of young controls (Figure 1f, Figure S2A,C). The number of slips was however only improved in the intermediate aged group tested on the round beam (Figure 1g), but not in the corresponding old age group tested on a square beam, or the old age group (Figure S2B,D). Collectively, these data demonstrate an age‐linked deterioration in gait and balance that can be mitigated by exercise.

### 2.2 Age, but not exercise affects the proprioceptive system

**FIGURE 2:**
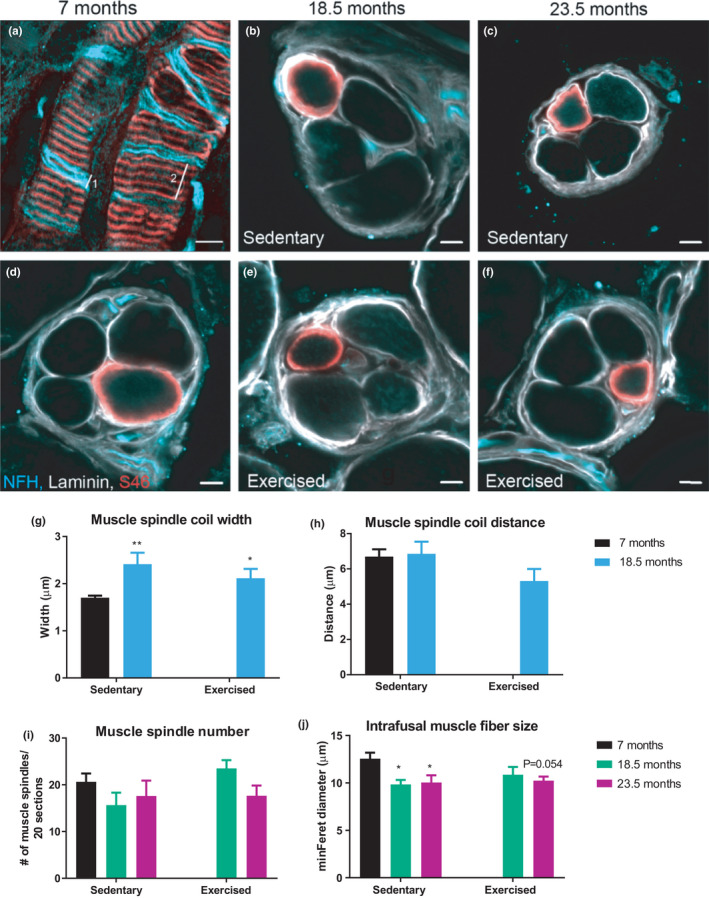
Age affects muscle spindle morphology. (a) Representative image of the longitudinal view of the muscle spindle receptor from EDL whole‐mount, and (b–f) cross‐sectional view from GS muscle. (g, h) Quantification of muscle spindle coil width (line 1 in panel 2a) (g) and coil distance (line 2 in panel 2a) (h) from whole‐mounted EDL muscle. *n* = 16 muscle spindles from a minimum five animals. (i) Number of muscle spindles per 20 serial sections, and H, the minimal Feret diameter of S46^+^ intrafusal muscle fibers. *n* = 2–3 muscle spindles per animal from a minimum five animals. Scale bar = 5 µm. Data show mean ± *SEM*. For panels (g) and (h), *p < 0.05; **p < 0.01 indicate statistically significant differences between 7 months controls and aged groups (unpaired, two‐tailed Student's *t*‐test). In panels (i) and (j), *p < 0.05 and *p*‐values indicates statistically significant differences and statistical trends between 7 months controls and aged groups (age effect) using a two‐way ANOVA comparison

To link the phenotypic gait and balance data to proprioceptive and vestibular circuits, we next studied components of the proprioceptive system. First, we used whole‐mounted fragments of the EDL muscle (Figure 2a) to evaluate sensory neuron coil morphology, and serial cross sections from GS muscle (Figure 2b–f) to assess muscle spindle number and diameter, respectively. Muscle spindle coil distance and width were measured in the equatorial region of the muscle spindle by scrolling through different images planes. The coil width of these type Ia proprioceptive sensory neurons (labeled with 1 in Figure 2a) was increased with age (Figure 2g), whereas the coil distance (labeled with 2 in Figure 2a) was unaffected (Figure 2h). Neither of these two parameters were affected by exercise. Second, while no change in muscle spindle number with age or exercise was detected (Figure 2i), the muscle spindle fiber diameter in the intermediate and old age groups was significantly smaller (Figure 2j). Even though exercise increased the diameter to values that were not significantly different from those of 7 months controls, this improvement was likewise not significantly different from the age‐matched sedentary counterparts (Figure 2j).

Next, to assess age‐ or exercise‐dependent effect on peripheral nerves, cross‐sections from the distal end of the sciatic nerve, supplying both sensory and motor innervation from and to hind limb muscles, were labeled for parvalbumin (PV) and laminin (Figure S3A–F). The number of PV^+^ nerve fibers was reduced in the old groups, however only reaching statistical significance in the trained mice (Figure S3G). The cross‐sectional diameter of PV^+^ fibers was neither affected by age nor exercise (Figure S3H). However, since PV^+^ is not an exclusive marker of proprioceptive sensory nerve fibers in the sciatic nerve, we also quantified the number of PV^+^ sensory neurons in cross‐sections of L4 dorsal root ganglia (DRGs) (Figure S3I). The absence of changes in the relative proportion of PV^+^/neuronal nuclear protein (NeuN)^+^ proprioceptive sensory neurons to total NeuN^+^ neurons in the DRG in all aging groups (Figure S3J) indicates that type Ia proprioceptive nerve endings in the periphery seem to be more vulnerable toward aging than neuronal number itself, as evidenced by increased muscle spindle coil width and decreased fiber size.

**FIGURE 3:**
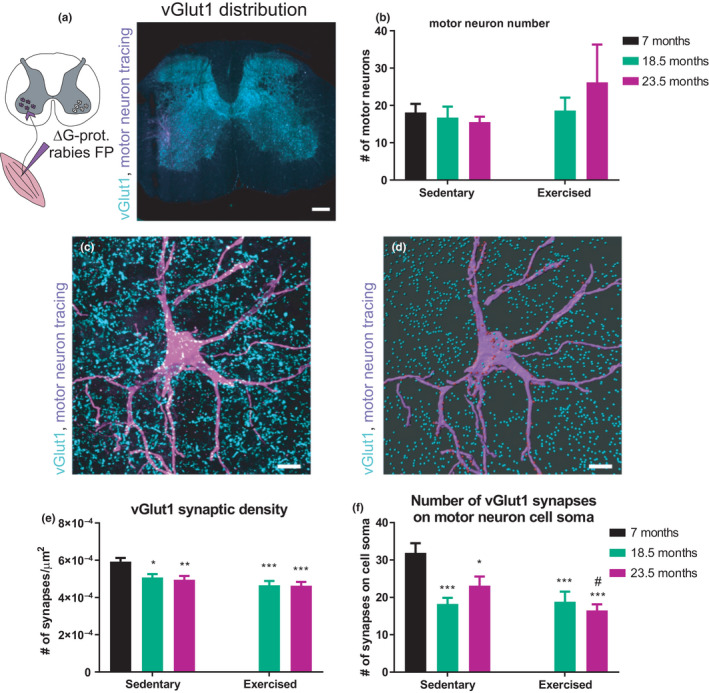
Proprioceptive input to motor neurons is decreased with age. Motor neurons from GS muscle were retrogradely traced using G‐protein‐deleted rabies viral vectors encoding fluorescent proteins (ΔG‐prot. rabies FP). (a) vGlut1 distribution in the lumbar region of a representative spinal cord section. B, Number of motor neurons of the GS motor neuron pool. A minimum of five animals per group was analyzed. (c, d) Representative example of a 3D projection of a motor neuron tree area (C) and the reconstructed version in Imaris (d). All reconstructed vGlut1+ synapses touching motor neurons are in red and the rest light blue. (e) Synaptic density (# synapses/µm^2^) was calculated by dividing the number of synapses touching the motor neuron by the surface area of the motor neuron. (f) Number of synapses making direct contact to motor neurons. A minimum of 20 motor neurons from a minimum of five different animals per group were analyzed. In (a) scale bar = 200 µm and in (c, d) scale bar = 100 µm. Data are displayed as mean with ±*SEM*. Using two‐way ANOVA, *p < 0.05; **p < 0.01; ***p < 0.001 indicate statistically significant differences between 7 months controls and aged groups (age effect), while ^#^ *p* < 0.05 indicates statistically significant differences between sedentary and exercised animals of the same age group (exercise effect)

Since type Ia proprioceptive sensory neurons have bifurcating axons, one projecting to the muscle spindles and the other projecting centrally to connect directly to motor neurons, we next investigated the effects of age and exercise on the axonal endings in the spinal cord. To do so, G‐protein‐deleted rabies viral vectors were used to retrogradely trace the GS motor neuron pool and spinal cord sections were labeled with the proprioceptive marker vesicular glutamate transporter 1 (vGlut1). Figure 3a shows the distribution of vGlut1 and the motor neuron localization on a representative spinal cord section. Next, we counted the number of motor neurons from the GS motor neuron pool and found no significant differences between groups (Figure 3b). This estimate of motor neuron numbers suggests that it is not likely that there is a significant neuronal loss. Spinal cord cross‐sections depicting GS motor neurons and co‐stained with vGlut1 were used to quantify proprioceptive synapses on this motor neuron pool. The number of vGlut1^+^ synapses on the motor neuron surface was normalized to the surface area of the reconstructed motor neuron to calculate the synaptic density (Figure 3c,d). We found that irrespective of exercise, the synaptic density was significantly reduced in both 18.5‐ and 23.5‐month‐old mice (Figure 3e). Furthermore, the number of synapses making direct contact to the soma of motor neurons was also diminished in an age‐dependent manner (Figure 3f). Consistently, we found fewer synapses between the cell soma and 60 µm distance from the dendrite root in both age groups relative to all vGlut1^+^ synapses (Figure S4A–D). Collectively, these data demonstrate an age effect on the type Ia proprioceptive system at both centrally projecting axons forming synaptic contacts to motor neurons as well as peripheral projecting axonal ends affecting muscle spindles that is insensitive to endurance exercise.

### 2.3 Exercise increases the vestibular input to motor neurons in old animals

**FIGURE 4:**
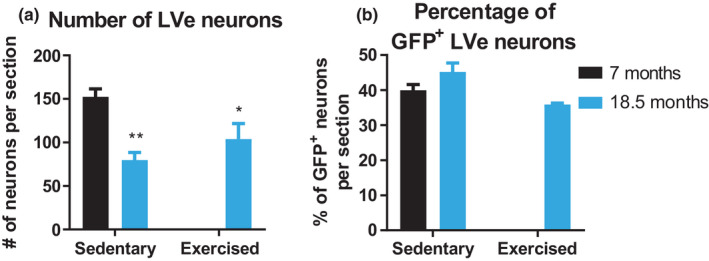
LVe neurons degenerate with age. (a) Quantification of NeuN^+^ LVe neurons per section. (b) Following injection of AAV vectors encoding synaptophysin‐GFP, the relative proportion of GFP^+^/NeuN^+^ to total NeuN^+^ neurons was determined. A minimum of three brain sections per animal was analyzed and the mean calculated. *n* = 5–7 animals. Scale bar = 100 µm. Data show mean ± *SEM*. *p < 0.05; **p < 0.01 indicate statistically significant differences between 7 months controls and aged groups (unpaired, two‐tailed Student's *t*‐test)

**FIGURE 5:**
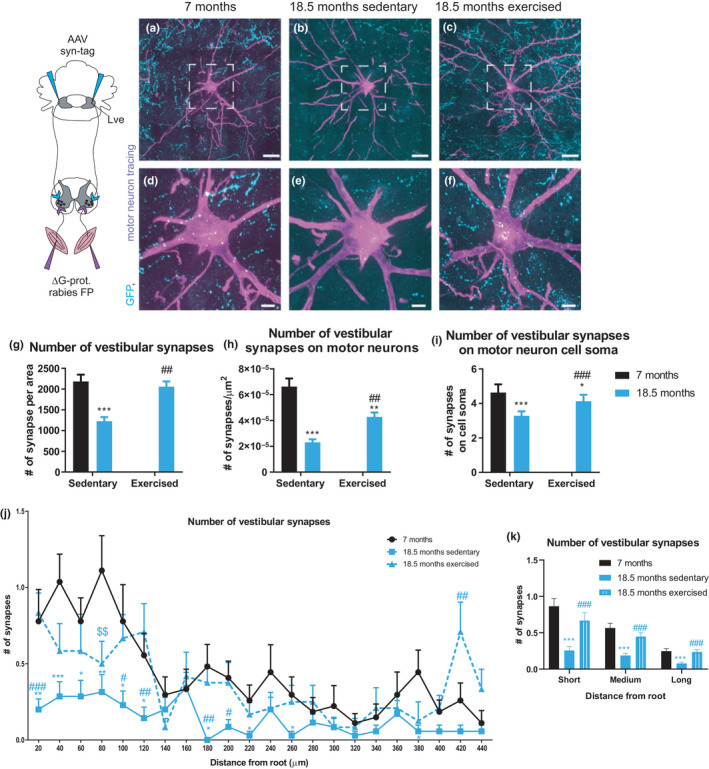
Exercise increases the vestibular input to motor neurons in old mice. Vestibular synapses were traced by injecting an AAV containing a plasmid with a synaptophysin‐GFP tag into the LVe. Motor neurons from soleus muscle were retrogradely traced using G‐protein‐deleted rabies viral vectors encoding fluorescent proteins (ΔG‐prot. rabies FP). (a–f) Representative images of motor neurons of 7 months (a, d), 18.5 months sedentary (b, e), and 18.5 months exercised (c, f) animals. G, Total number of vestibular synapses per motor neuron area. (h) Vestibular synaptic density (# synapses/µm^2^) that was calculated by dividing the number of synapses touching the motor neuron by the surface area of the motor neuron. (i) Number of synapses on the motor neuron cell soma. (k) Number of vestibular synapses on the motor neuron cell soma within short (20–60 μm), medium (80–200 μm) and long (220–440 μm) distance from the root. *p < 0.05; **p < 0.01; ***p < 0.001 indicate statistically significant differences between 7 months control and aged groups.^##^ *p* < 0.01; ^###^ *p* < 0.001 indicate statistically significant differences between sedentary and exercised animals of the same age group (unpaired, two‐tailed Student's *t*‐test). (j) Synaptic distribution along motor neuronal dendrite branches measured in distance from root. *p < 0.05; **p < 0.01; ***p < 0.001 indicate statistically significant differences between 7 months control and aged sedentary groups. ^$$^ *p* < 0.01 indicates statistically significant differences between 7 months control and aged exercised groups. ^#^ *p* < 0.05; ^##^ *p* < 0.01; ^###^ *p* < 0.001 indicate statistically significant differences between sedentary and exercised animals of the same age group (unpaired, two‐tailed Student's *t*‐test). A minimum of 41 motor neurons from a minimum of five different animals per group were analyzed. In (a–c) the scale bar = 100 µm and in (d–f) 50 µm. Data are displayed as mean with ±*SEM*

Our results pertaining to proprioception suggest that endurance exercise likely improves balance and gait by affecting other circuits, for example, the vestibular system. Since aging is linked to neurodegeneration (Chakrabarti & Mohanakumar, 2016), we first quantified the number of neurons in the LVe (Figure 4a), by creating an image stack and counting all LVe neurons on the different image planes (representative images of young and old mice depicted in Figure 4b,c). We observed a reduced number of neurons in these nuclei, which was not affected by exercise (Figure 4d). Next, we traced vestibular monosynaptic contacts to motor neurons by anterogradely tracing descending vestibular axons with an adeno‐associated viral (AAV) vector encoding a synaptophysin‐green fluorescent protein (GFP)‐tagged fusion protein into the LVe. A quantification of GFP^+^/NeuN^+^ neurons compared to total NeuN^+^ neurons (Figure S5A–C) revealed similar proportions of injected cells in all conditions (Figure 4e). In the spinal cord, GFP^+^ nerve terminals and retrogradely traced motor neurons innervating the soleus muscle using g‐protein deleted rabies virus were measured (Figure 5a–f and Figure S5D–F depicting the distribution of vestibular synapses and soleus motor neurons in representative spinal cord sections). Old sedentary animals exhibited a significant reduction in the overall number of vestibular synapses (Figure 5g), fewer synapses of descending vestibular axons making contact to the soleus motor neuron pool (Figure 5h) and to the respective cell soma (Figure 5i) compared to 7 months controls. In stark contrast, the total number of vestibular synapses, as well as the number of synapses on the surface and the soma of the soleus motor neurons was significantly higher in old exercised mice compared to their sedentary counterparts (Figure 5g–i). These effects of age and exercise were most prominent at close distances from the motor neuron dendrite root (Figure 5j,k, Figure S6A,B). Taken together, exercise rectifies the age‐associated decline in overall vestibular nerve terminals and in the number of vestibular synaptic contacts to specific motor neuron pools, without affecting neurodegeneration of vestibular neurons in the LVe.

## 3 DISCUSSION

**FIGURE 6:**
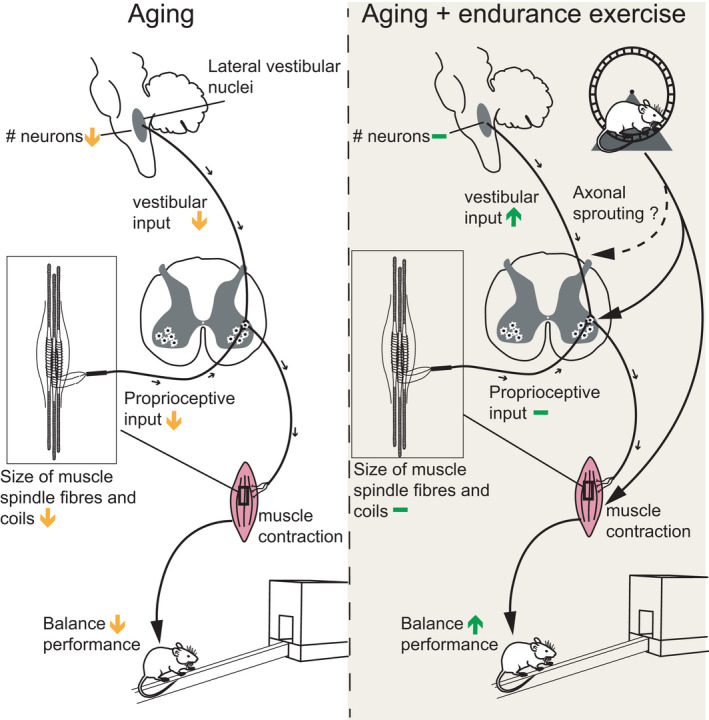
The effects of age and exercise on proprioceptive and vestibular input into motor neurons. Aging results in a deterioration of the various components of the proprioceptive and the vestibular systems. Endurance exercise counteracts vestibular deterioration by increasing vestibular synaptic input into specific motor neuron pools. In combination with the beneficial effects of training on the neuromuscular junction and skeletal muscle, balance performance in old mice is improved

Loss of balance, aberrant posture, and altered gait are important factors that contribute to the functional deterioration of skeletal muscle in sarcopenia (Fuller, 2000). Moreover, the increased risk of falls that is precipitated by these pathological changes strongly drives muscle mass loss as a results of the ensuing avoidance of physical activity due to insecurity as well as disuse based on a sedentary lifestyle, immobilization and hospitalization (Khanuja et al., 2018). A better understanding of the processes that control balance in aging is therefore instrumental to mitigate the dramatic loss in quality of life, and the increase in morbidity and mortality of elderly individuals. Besides a well‐described effect of age on neuromuscular junctions, it is unclear how balance and coordination are controlled on the neuronal level in old mice and humans. In this manuscript, we now for the first time have studied in‐depth the neuronal circuits of the proprioceptive and vestibular system that are the predominant regulators of balance, and linked these findings to a comprehensive phenotypic assessment of gait and balance in mice at different ages and in a sedentary and trained state, respectively (Figure 6). While we observed age‐associated degenerative events in both systems, we discovered a surprising and marked effect of endurance exercise to alleviate the age‐linked degeneration in synaptic vestibular input to specific motor neuron pools, for some parameters to levels that are indistinguishable from young control mice. Interestingly, this exercise‐mediated boost in vestibular synapses in the spinal cord occurred in the absence of a protective effect of training on the loss of neurons in the LVe. In addition to degeneration of LVe neurons with age, which has also been previously reported in mice (Sturrock, 1989) and humans (Diaz et al., 1993), decreased regeneration of LVe axons has been found after spinal cord lesion (Roozbehi et al., 2015), suggesting a high vulnerability of LVe axons to damage. On a functional level, vestibular input to motor neurons increased concomitantly with improved balance in exercised old mice, indicating that elevated levels of vestibular synapses on motor neurons could likely have promoted increased balance capacity, even in the absence of any beneficial effect of proprioceptive connectivity. These findings support a model in which endurance exercise could promote the regeneration of descending vestibular efferents by promoting axonal sprouting and synapses formation compensating for the decline in overall neuronal number in the LVe. Such an exercise‐induced axonal sprouting in aging would be in line with observations that treadmill training promotes axonal sprouting leading to improved motor recovery after spinal cord hemi‐section (Goldshmit et al., 2008). The mechanistic basis of our results on vestibular connectivity or the axonal sprouting in spinal cord lesions is unknown, but could involve neurotrophic factors. First, exercise increases neurotrophic factors in muscle and spinal cord (English et al., 2014; Gomez‐Pinilla et al., 2001; Molteni et al., 2004). Second, the brain‐derived neurotrophic factor (BDNF), neurotrophin‐3 (NT3) and glial cell‐derived neurotrophic factor (GDNF) promote axonal recovery and increase synapse formation in spinal cord‐injured animals (Deng et al., 2013; Ye & Houle, 1997). Future studies thus will aim at investigating the regulation and role of neurotrophic factors in motor neuron innervation by the vestibular system with age and exercise.

Similar to the vestibular system, age was strongly linked to deleterious effects on the muscle spindles and proprioceptive input to motor neurons. Interestingly, most of the age‐associated changes were found at the axonal terminals of the type Ia proprioceptive sensory neurons. First, the muscle spindle exhibited altered morphologies, similar to other data in human (Liu et al., 2005; Swash & Fox, 1972) and rodent studies (Kim et al., 2007; Vaughan et al., 2017), the exact functional consequence of which are currently unknown. Second, the reduced connectivity of proprioceptive sensory neurons to motor neurons in the spinal cord, similar to that observed in aged monkeys (Maxwell et al., 2018), implies an age‐dependent process of synaptic elimination in the absence of an effect on proprioceptive neuronal number in the DRG. However, unlike the vestibular system, no effect of endurance training was found on any of these pathological changes in old mice. These data suggest that in contrast to vestibular wiring, type Ia proprioceptive connectivity is insensitive to endurance exercise, at least at old age. It is conceivable that the training paradigm might be important to differentially modulate the vestibular and the proprioceptive systems, and interventions that differ in intensity and/or volume, or resistance training would lead to a different outcome. Moreover, it is possible that other descending pathways also relaying vestiublar information could be involved in the observed improvement in balance of aged trained mice. Finally, other fibers that carry proprioceptive information, for example, type Ib and type II proprioceptive neurons, could also be affected by age and/or exercise.

In summary, we now provide a complete overview on the effects of age and endurance exercise on proprioceptive and vestibular connectivity to motor neurons. Even though only vestibular connectivity was ameliorated by exercise in this context, these changes likely are potent enough to at least contribute to the dramatic improvement in gait and balance of old, trained mice. Of note, generic endurance exercise, and not specific balance training, only performed at old age, at moderate dose (3 times per week), and for different times (6 or 12 weeks), at two different volumes (1 h or 45 min) was sufficient to elicit these changes, at least when combined with free wheel running access. It will be interesting to compare this outcome on neuronal connectivity and phenotypic adaptation to life‐long training studies, and to investigate the persistence of the effects upon training cessation. Unfortunately, until the underlying molecular mechanisms are elucidated, pharmacological targeting will remain difficult. However, our results highlight the importance to promote physical activity in the elderly, a population that requires motivation, encouragement, education and assistance to start and adhere to and complete training programs (Hughes et al., 2019). If properly implemented and supported, such interventions would not only benefit the elderly individuals, but also reduce the exploding burden on health care systems by improving independence, morbidity and mortality.

## 4 MATERIALS AND METHODS

### 4.1 Animals

All animal experiments were done with male C57BL/6J mice that were obtained from Janvier. Animals were fed ad libitum with regular chow diet and kept under a 12‐h/12‐h light–dark cycle at 23°C. All experiments were performed in accordance with the Swiss federal guidelines for animal experimentation and were approved by the Kantonales Veterinäramt of the Kanton Basel‐Stadt. Animals were randomized, and experimental groups were generated as indicated in Figure 1a.

### 4.2 Exercise protocols

Mice aged 15.5 and 20.5 months were trained on a treadmill for 12 weeks, 3 times per week for 1 h. Additionally, 17‐month‐old mice were trained on a treadmill for 6 weeks, 3 times per week for 45 min. All exercising animals had free access to running wheels during the intervention period. The treadmill speed was determined prior to the intervention by testing the maximum speed of the animals. Accordingly, the training speed was gradually increased from 50% to 80% maximum speed at an angle of 5° or until mice could not keep up the pace during training sessions.

The protocol to assess the maximum speed was set at 8 m/min for 3 min and was increased every 2 min by 2 m/min at an angle of 5° until the mice were exhausted.

### 4.3 In vivo testing

Unless otherwise stated, all in vivo tests were done 4 days before, 6 weeks after, and 12 weeks after the start of the exercise intervention, respectively. When animals were exercised for 6 weeks, in vivo test were done 4 days before and 6 weeks after the intervention.

### 4.4 Balance beam test

Mice were familiarized to cross an inclined beam leading to a red box 1 day before the testing in three practice trials. The time to cross the beam and the number of slips were measured to assess balance performance. The intermediate and old age group were tested on a round balance beam system with while the 6‐week‐exercise group was tested on a square beam. Thus for the 6‐week‐exercise group, an additional control group aged 7 months was used for baseline testing.

### 4.5 Gait analysis

The gait of mice was assessed using the CatWalk XT system by Noldus. Briefly, mice walked voluntarily across a glass platform. Several paw print sequences were captured by a camera located under the platform and gait parameters were calculated using the Noldus software.

### 4.6 Proprioceptive tracing

Motor neuron pools from gastrocnemius muscles were retrogradely traced using a G‐protein deleted rabies virus as described previously (Basaldella et al., 2015). Briefly, GS and soleus muscles of anesthetized mice were intramuscularly injected with virus. After 4 days of incubation, mice were anesthetized and transcardially perfused. Injection specificity was visually confirmed after dissection of the muscle. Unspecific injections were excluded from analysis.

### 4.7 Vestibular tracing

To trace descending vestibular monosynaptic contacts to motor neurons, an AAV containing a plasmid with a synaptophysin‐GFP‐Tag fusion protein was bilaterally injected into the LVe as described (Basaldella et al., 2015). Briefly, AAV‐virus was injected into the LVe using the coordinates 0.24 mm antero‐posterior, 0.134 mm medio‐lateral and 0.355 mm dorso‐ventral from lambda. The virus was incubated for 14 days, after which the animals were perfused. 4 days before perfusion, the soleus muscle was injected with G‐protein deleted rabies virus to trace soleus motor neuron pools.

### 4.8 Immunohistochemistry

Spinal cord, DRG and sciatic nerve: Mice were anesthetized and transcardially perfused with 20 ml saline solution followed by 70 ml 4% PFA in phosphate buffer. After perfusion, spinal cords, DRGs and sciatic nerves were dissected and post‐fixed overnight in 4% PFA. After fixation, the samples were sucrose‐protected for 24 h in 30% sucrose in PBS and then frozen and cut in a cryostat. Spinal cord sections of 60 µm thickness were blocked 1 h in 4% BSA and 5% goat serum in 0.5% Triton‐X‐100 at room temperature and then incubated overnight in primary antibody at 4°C followed by three washes in PBS. Spinal cord sections were incubated overnight in secondary antibody at 4°C and then washed three times with PBS before sections were mounted. DRGs and sciatic nerves were cross‐sectioned at 10‐µm thickness and were blocked for 1 h in 4% BSA and 5% goat serum in 0.5% Triton‐X‐100 at room temperature and then incubated overnight in primary antibody at 4°C followed by three washes in PBS. Then the sections were incubated for 1 h in secondary antibody and washed three times with PBS before mounting.

*Brains*: Mice were anesthetized and transcardially perfused with 20 ml saline solution followed by 70 ml 4% PFA in PB buffer. After perfusion, brains were dissected and post‐fixed overnight in 4% PFA and stored in PBS until sectioning. Brains were cut at 40 µm on a vibratome and blocked for 1 h in 4% BSA and 5% goat serum in 0.5% Triton‐X‐100 at room temperature and then incubated for two nights in primary antibody at 4°C. Sections were washed three times in PBS and incubated in secondary antibody at RT for 1 h. After three washes with PBS, brain sections were mounted with mounting medium from Vecta shield on slides.

*Skeletal muscle sections*: Muscles were fresh‐frozen in isopentane cooled in liquid nitrogen. To assess muscle spindles, 60 µm cross‐sections from gastrocnemius muscle were blocked 1 h in 4% BSA and 5% goat serum in 0.5% Triton‐X‐100 at room temperature and then incubated overnight in primary antibody at 4°C followed by three washes with PBS. Muscle sections were incubated for 1 h in secondary antibody and washed three times with PBS before mounting.

*Skeletal muscle whole mounts*: EDL muscle was dissected and separated by their tendons into four fragments. EDL fragments were fixed in 4% PFA in PB buffer for 20 min and then washed three times followed by blocking in 4% BSA and 5% goat serum in 0.5% Triton‐X‐100. Then, EDL fragments were incubated in primary antibody for 4 days at 4°C and then washed three times in PBS. After, EDL fragments were incubated for 2 days in secondary antibody at 4°C and then washed three times before whole‐mounting on slides. All antibodies used are listed in Suppl. Table S1.

### 4.9 Microscopy and image analyses

*Motor neurons*: 20 tiles and stacks of the motor neuron tree were imaged on a spinning disc Nikon eclipse connected to a Visitron system with a 63× objective. For the proprioceptive tracing, we imaged stacks of 1 µm sampling. For the vestibular tracing, stacks with 300 nm sampling were chosen. Stacks and tiles were stitched together in Fiji. The motor neuron surface was reconstructed in Imaris and synapses were detected by the Imaris spot detection function. A Matlab extension tool was used to calculate the number of spots touching the reconstructed motor neuron. The number of such spots was normalized to the motor neuron surface area. In the proprioceptive tracing, an area of 650 µm by 650 µm centered on the motor neurons was evaluated. γ‐Motor neurons were discriminated from α‐motor neurons based on their size and were excluded from analysis. The synapse distribution on the motor neuron tree was analyzed using Neurolucida. A blinded observer manually traced the outline of the motor neuron soma and the dendrites. All synapses touching the motor neuron were marked and the software calculated the distance of this mark to the motor neuron soma. The number of synapses contacting directly the cell body were counted manually. For analyses, a minimum of three motor neurons per animal were assessed.

*Muscle spindles*: To visualize muscle spindles with the sensory nerve, muscle cross section and EDL whole mounts were labeled for myosin heavy chain S46 (S46), laminin, DAPI and neurofilament H (NFH). The slow‐tonic myosin heavy chain S46 is most likely expressed in nuclear bag1 and bag2 intrafusal muscle fibers, and usually stains one fiber per spindle (Vaughan et al., 2017). Muscle spindles were thus identified by S46 positive intrafusal muscle fibers surrounded by a capsule, nuclear aggregation within intrafusal muscle fiber and a NFH staining near intrafusal muscle fibers. Muscle spindles from GS cross sections from the upper half of the muscle were imaged on a LSM 700 confocal system from Zeiss with a 63x objective. Muscle spindle number was counted per 20 serial sections and the minimum Feret diameter of S46 positive intrafusal muscle fibers was measured in Fiji. Sections from at least four animals per group were assessed, 2 to 6 muscle spindle sections were analyzed per animal and the average calculated. Stacks of muscle spindles from EDL whole mounts were imaged on a LSM 880 confocal system from Zeiss with a 63x objective and 0.75 µm sampling. Muscle spindle coil distance and width were measured in Fiji by scrolling through the individual images of the stack. Coil width and distance were measured at the equatorial region of intrafusal muscle fibers as evidenced by nuclear aggregation. Type II endings were excluded from analysis. The average coil distance and width for each muscle spindle was calculated by measuring at least two coils. Overall, a minimum of two muscle spindles from at least five animals per group were assessed.

*Sensory neurons*: L4 DRG sections were imaged with an AxioScan.Z1 slide scanner from Zeiss using a 20x objective. Proprioceptive sensory neurons were identified by PV^+^ cells. All sensory neurons were labeled by NeuN. The number of PV and NeuN positive sensory neurons was counted manually by taking the average of minimum two sections from the same animal. A distance of at least 30 µm between individual sections was used for the analysis.

*Sciatic nerves*: Cross sections from sciatic nerves were imaged with an AxioScan.Z1 slide scanner from Zeiss using a 20× objective. Sections were labeled for PV and laminin. The number of PV^+^ axons was counted manually. The average of minimum two sections per animal was calculated.

*Brain sections*: Coronal brain sections were imaged on a spinning disk Nikon eclipse connected to a Visitron system with a 63x objective. To visualize neurons from the lateral vestibular nucleus, sections were stained for NeuN and anti‐GFP. To capture the whole vestibular nuclei area, 12 by 12 tiles were imaged. The lateral vestibular nucleus was identified as the region that includes large cells between the 4th ventricle and the inferior cerebellar peduncle, in line with other anatomical and morphological outlined in the Allen Mouse Brain Reference Atlas (https://mouse.brain‐map.org/). Using several tiles and stacks with 0.8 µm sampling, for each brain section the number of GFP^+^ and the total number of neurons in the lateral vestibular nucleus was counted manually by scrolling through the different image planes. The average neuron number from at least three different sections per animal was used to calculate an average neuron number per section from a minimum of five different animals.

### 4.10 Statistical analysis

Unless otherwise stated, significance between groups was assessed using two‐way ANOVA followed by a Sidak multiple comparison test or unpaired, two‐tailed Student's *t*‐test was performed as described in the figure legends. In all figures, the mean value and *SEM* were used to plot the data with error bars.

## CONFLICT OF INTEREST

The authors have no conflict of interest related to this manuscript.

## AUTHOR CONTRIBUTIONS

FB and CH involved in conceptualization and experimental design. FB, SS, GR, ACD, and KRT performed the experiments. FB, ACD, KRT, and CH analyzed and interpreted the data. GR, ACD, and KRT provided tools and methods. FB and CH wrote the manuscript. CH involved in funding acquisition and project supervision.

## Supporting information

Appendix S1

Video S1A

Video S1B

## Data Availability

The data that support the findings of this study are available from the corresponding author upon reasonable request.
