# Supplementary material for: Exercise‐linked improvement in age‐associated loss of balance is associated with increased vestibular input to motor neurons
Source: Aging Cell. 2020 Nov 11;19(12):e13274. doi: 10.1111/acel.13274 (PMC7744958; doi:10.1111/acel.13274)
Supplement: Supplementary file 1 — Appendix S1 [file ACEL-19-e13274-s001.pdf]

Supplemental data

Content:

Supplemental Figures S1-S6

Supplemental Table S1

Supplemental Movies S1 (Catwalk gait analysis) and S2 (Balance beam analysis)

Supplemental Figures

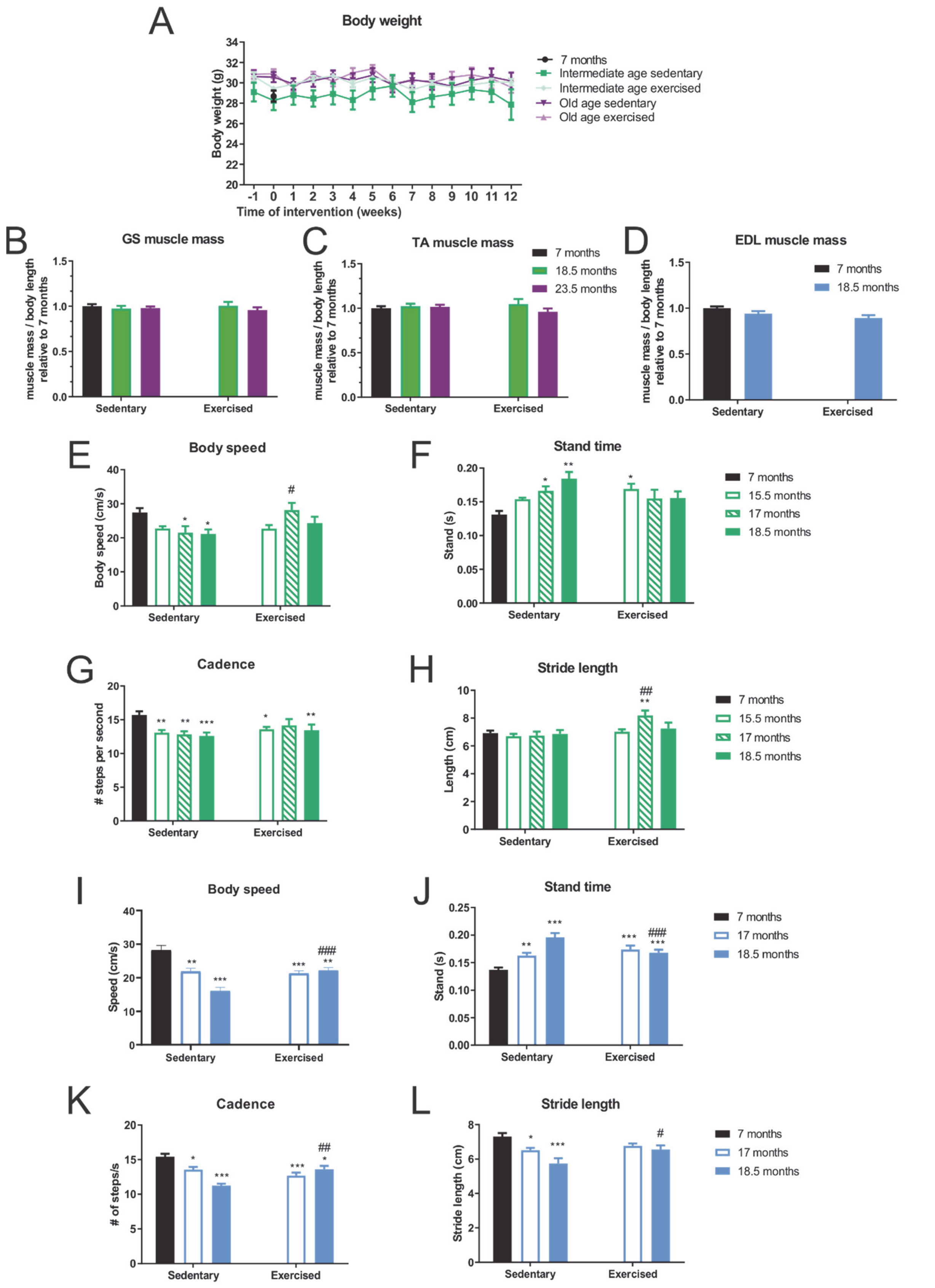

Suppl. Fig. S1. Age and exercise effects on gait. A, Body mass of 7 months control, intermediate and old age intervention groups. **B-D,** Mass of GS (**B**), TA (**C**) and EDL

(**D**) muscles of 7 months controls and aged sedentary and exercised mice normalized to body length. **E-L,** Catwalk gait analysis. Mice voluntarily walked across a glass platform where paw prints were recorded and gait was assessed: body speed (cm/s) (**E**, **I**), stand time (s) (**F**, **J**), cadence (steps/s) (**G**, **K**), and stride length (cm) (**H**, **L**). Data are displayed as mean with ± SEM. n= 6 to 9 animals per group. In two-way ANOVA; $^{*}p < 0.05$; $^{**}p < 0.01$; $^{***}p < 0.001$ indicate statistically significant differences between 7 months controls and aged groups (age effect) while $^{\#}p < 0.05$; $^{\#\#}p < 0.01$; $^{\#\#\#}p < 0.001$ indicate statistically significant differences between sedentary and exercised animals of the same age group (exercise effect).

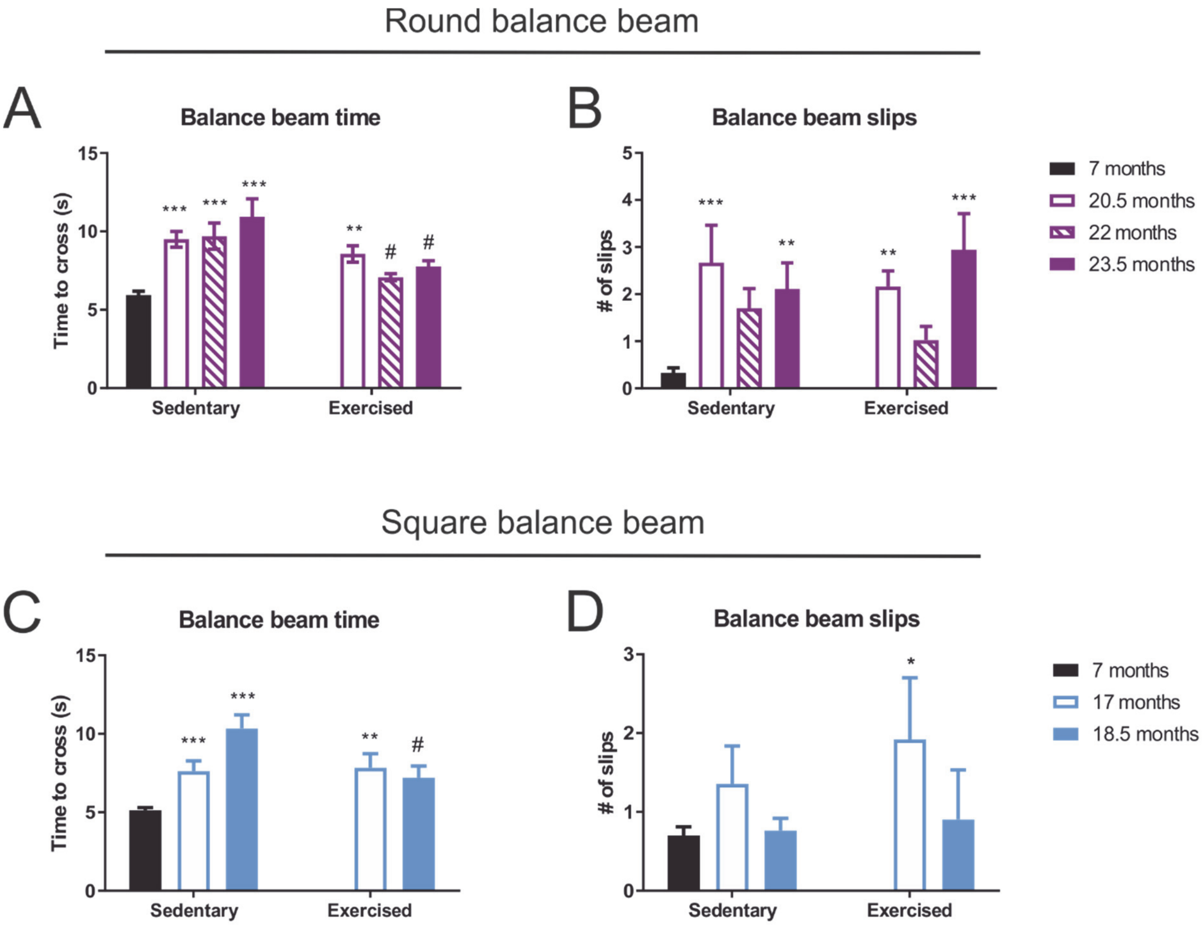

Suppl. Fig. S2. Exercise improves balance in old mice. Balance of aged exercised and sedentary mice was assessed using a balance beam test with two different beams. **A**, Time to cross the length of a round balance beam. **B,** Number of slips while traversing a round balance beam in old age animals. **C,** Time to cross the length of a square balance beam. **D,** Number of slips while traversing a square balance beam. Data are displayed as mean with ± SEM. n= 6 to 9 animals per group. In two-way ANOVA, *p < 0.05; **p < 0.01; ***p < 0.001 indicate statistically significant differences between 7 months controls and aged groups (age effect) while $^{\#}p < 0.05$ indicates statistically significant differences between sedentary and exercised animals of the same age group (exercise effect).

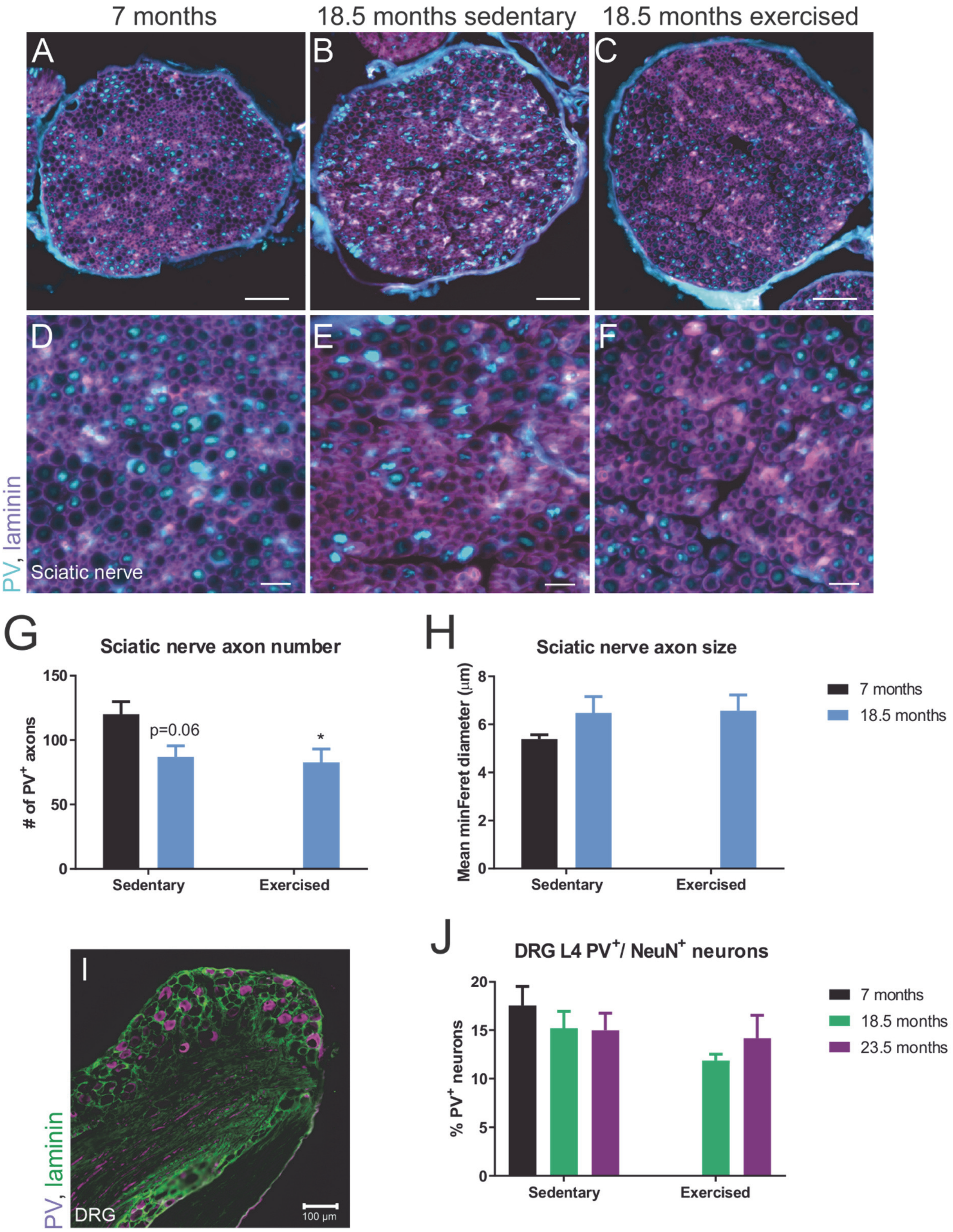

Suppl. Fig. S3. Proprioceptive sensory neuron number is unaffected by age or exercise. A-F, Representative images of sciatic nerve cross-sections labelled for PV in cyan and laminin in purple. **G,** Number of PV+ nerve fibres. **H,** Minimal Feret diameter of PV+ fibers. **I**, Representative image of a section from L4 DRGs. **J**, $PV^+/NeuN^+$ proprioceptive sensory neurons were counted and the ratio relative to the

total number of sensory NeuN$^+$ neurons was calculated. Scale bar = 50 μm unless indicated. Data show mean ± SEM. *$p < 0.05$ indicates statistically significant differences between 7 months old controls and aged groups (unpaired, two-tailed Student's T-test). Trends are indicated by stating the P-values relative to 7 months old controls.

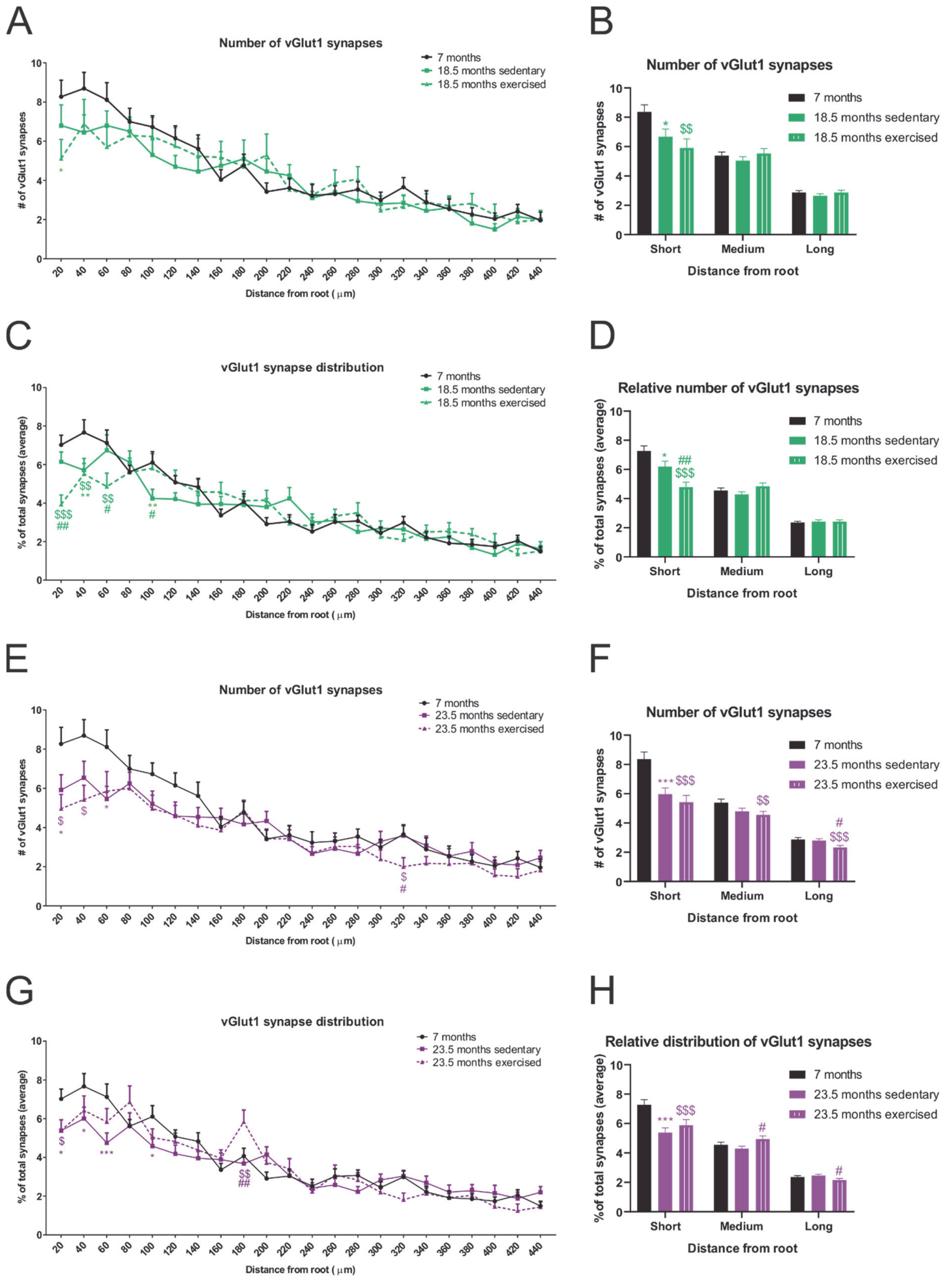

Suppl. Fig. S4. Loss of vGlut1 synapses is primarily proximal. Motor neurons from GS muscle were retrogradely traced using G-protein deleted rabies viral vectors. **A-H,** vGlut1^{+} synaptic distribution along motor neuronal dendrite branches measured in distance from root in absolute numbers (**A**, **E**) and relative to total vGlut1 synapse

count (**C**, **G**). Number of vestibular synapses on the motor neuron cell soma within short (20-60 µm), medium (80-200 µm) and long (220-440 µm) distance from the root in absolute numbers (**B**, **F**), and relative to total vGlut1 synapse count (**D**, **H**). Data are displayed as mean with ± SEM. n= 6 to 9 animals per group. *$p < 0.05$; **$p < 0.01$; ***$p < 0.001$ indicate statistically significant differences between 7 months controls and aged sedentary groups. $^{\$}p < 0.05$; $^{\$\$}p < 0.01$; $^{\$\$\$}p < 0.001$ indicate statistically significant differences between 7 months controls and aged exercised groups. $^{\#}p < 0.05$; $^{\#\#}p < 0.01$ indicate statistically significant differences between sedentary and exercised animals of the same age group (unpaired, two-tailed Student's T-test).

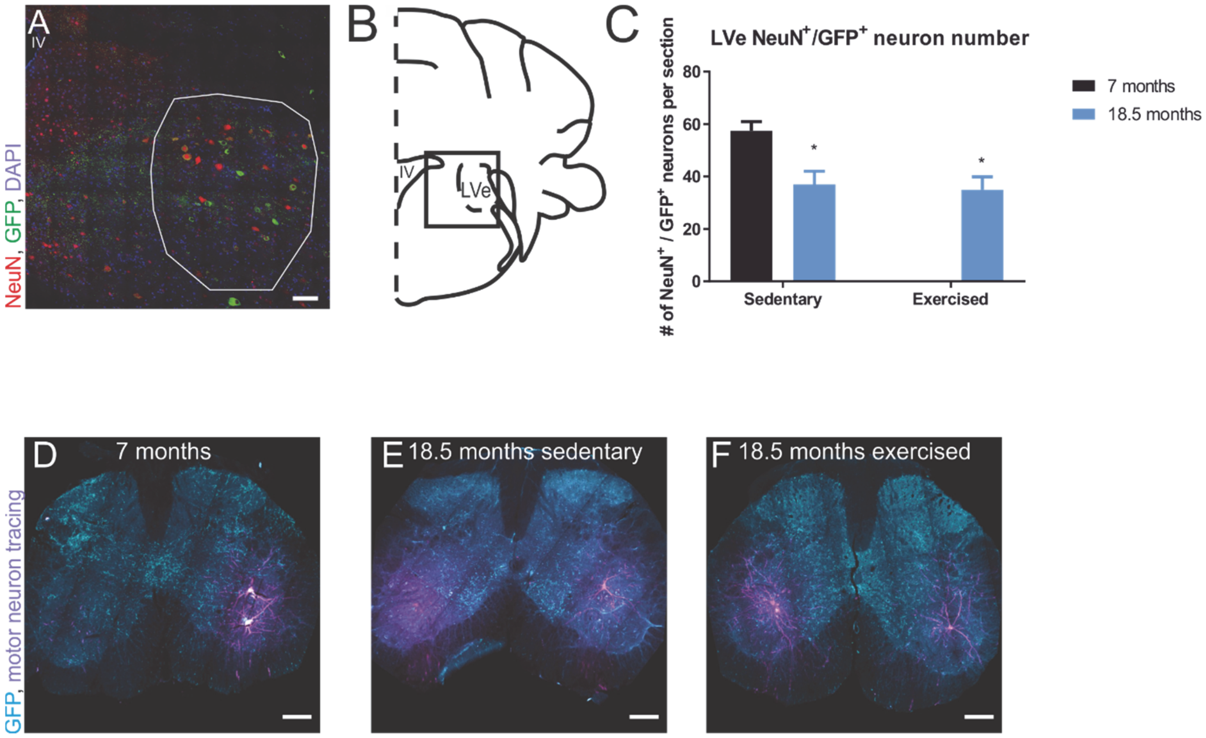

Suppl. Fig. S5. AAV-mediated tracing of descending LVe vestibular neurons. An AAV-vector containing a plasmid with a synaptophysin- GFP tag was injected into the LVe. **A,** Representative image of LVe region from coronal brain section labelled for NeuN in red, GFP in green, and DAPI in blue. **B,** Schematic representation of coronal brain section with IV ventricle and the LVe region (dotted line). The square represents the field of view of the images. **C,** Number of infected LVe neurons per section. A minimum of three brain sections per animal were analysed and the mean calculated. n = 5 to 7 animals. **D-F**, Distribution of vestibular synapses and motor neurons in representative spinal cord sections. In A scale bar = 100 µm and in D to F scale bar = 200 µm. Data show mean ± SEM. *$p < 0.05$ indicates statistically significant differences between 7 months controls and aged groups (unpaired, two-tailed Student's T-test).

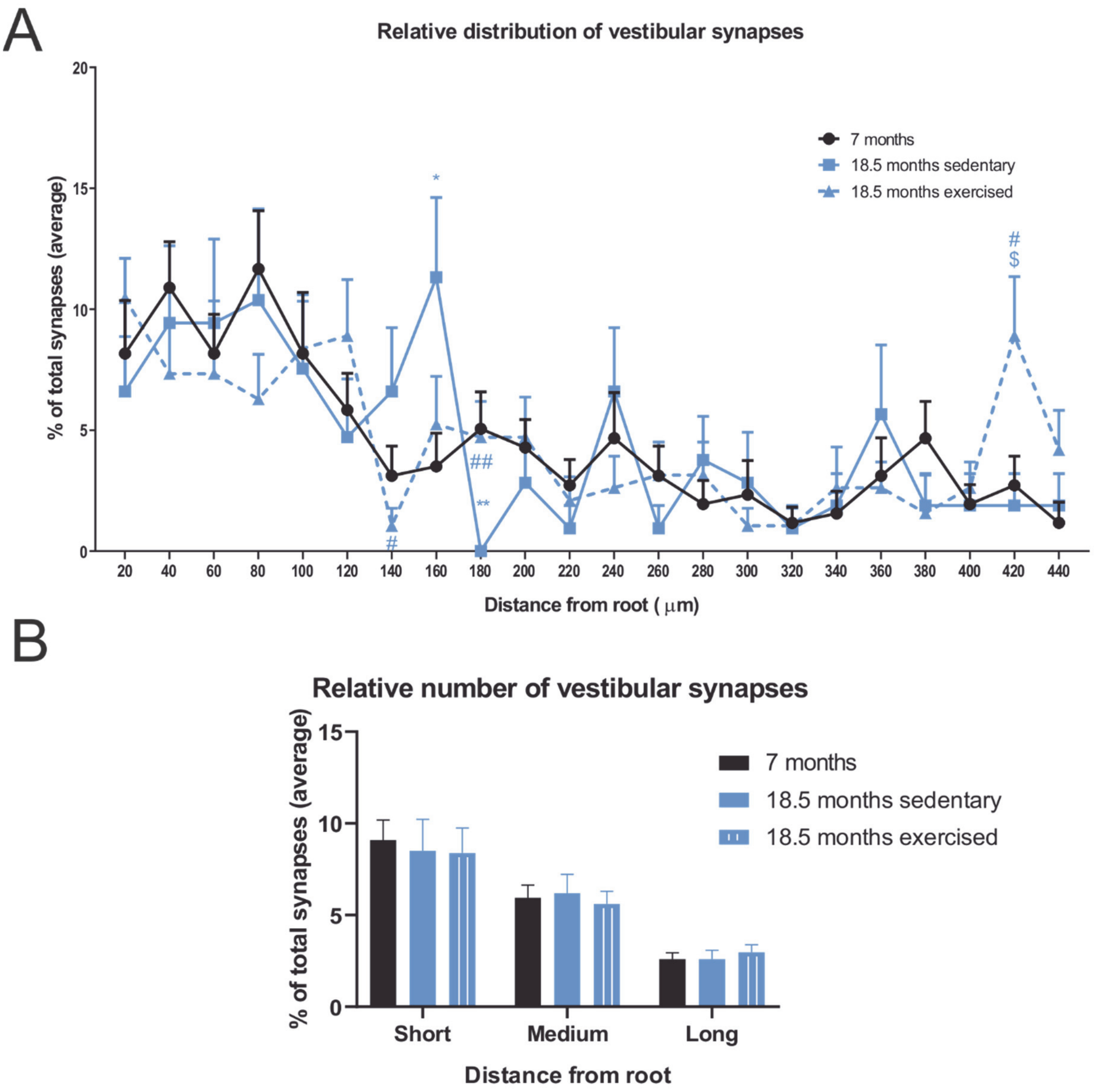

Suppl. Fig. S6. The relative distribution of vestibular synapses is largely unchanged. Vestibular synapses were traced by injecting an AAV containing a plasmid with a synaptophysin-GFP tag into the LVe. Motor neurons from soleus muscle were retrogradely traced using G-protein-deleted rabies viral vectors encoding fluorescent proteins (ΔG-prot. rabies FP). **A**, Relative number of vestibular synapses on the motor neuron cell soma. **B**, Number of vestibular synapses on the motor neuron cell soma within short (20-60 μm), medium (80-200 μm) and long (220-440 μm) distance from the root. A minimum of 41 motor neurons from a minimum of five different animals per group were analyzed. *p < 0.05; **p < 0.01 indicate statistically significant differences between 7 months control and aged sedentary groups. $^{\$}p < 0.05$ indicates

statistically significant differences between 7 months control and aged exercise groups. $^{\#}p<0.05$; $^{\#\#}p < 0.01$ indicate statistically significant differences between sedentary and exercised animals of the same age group (unpaired, two-tailed Student's T-test).

Suppl. Table S1: List of all antibodies used for immunohistochemistry.

Target	Dilution	Reference	Company
Anti-Laminin alpha 2	1:500	ab11576	abcam
Anti-S46	1:50		Developmental Studies Hybridoma Bank
Anti-NFH	1:200	AB1991	Chemicon
Anti-Parvalbumin	1:500	PV27	Swant
Anti-NeuN	1:500	ab134014	abcam
Anti-NeuN	1:100	MAB377	Millipore
Anti-vGlut1	1:20'000	AB5905	Chemicon
Anti-RFP	1:5'000	600-401-379	Rockland
Anti-GFP	1:1'000	A10262	Invitrogen
A488 anti-rat	1:100	A11006	Invitrogen
A568 anti-mouse	1:100	A21124	Invitrogen
A647 anti-rabbit	1:100	711-605-152	Jackson Immuno Research
A568 anti-rabbit	1:100	A10042	Invitrogen
A647 anti-guinea pig	1:100	AP193SA6	Chemicon
A488 anti-chicken	1:100	A11039	Invitrogen
